# Flowing Fibers: Subsurface Sampling Is Key to Understanding Natural and Plastic Textile Fiber Pollution in Rivers

**DOI:** 10.1021/acsestwater.5c00998

**Published:** 2025-12-29

**Authors:** Harley Nicholls, Catherine Sanders, David B. Ryves, Edwin Baynes, Kelly J. Sheridan, Thomas Stanton

**Affiliations:** † Department of Geography and Environment, 5156 Loughborough University, Loughborough LE11 3TU, U.K.; ‡ Department for Environment, Food and Rural Affairs (Defra), 41726 Environment Agency (EA), Bristol BS1 5AH, U.K.; § School of Natural Sciences, 4547 University of Lincoln, Brayford Way, Brayford Pool, Lincoln LN6 7TS, U.K.; ∥ Department of Applied Sciences, Faculty of Health and Life Sciences, 5995 Northumbria University, Newcastle Upon Tyne NE1 8ST, U.K.

**Keywords:** microplastic transport, microplastic monitoring, textile fibers, natural fibers, microfibers, microplastic methods, aquatic pollution, experimental flume

## Abstract

There is a pressing need to understand the pathways of textile fibers as anthropogenic pollutants in the environment. Current efforts to understand textile fiber pollution in waterways have relied on surface-sampling methodologies without consideration for environmental heterogeneity. Moreover, how nonplastic textile fibers behave in the environment is not known. Here, for the first time, we experimentally quantify the role that fiber type (cotton, wool, polyester, and acrylic) and riverbed roughness (flat, fine gravel, and coarse gravel) have on the vertical distribution of transported fibers using an experimental, recirculating flume. Analysis of the vertical profile distributions of 18,793 cotton, wool, polyester, and acrylic fibers indicated that bed substrate significantly altered fiber transport pathways, which was consistent across all tested fiber types. Our findings indicate that surface-only sampling will substantially under-record fiber fluxes, but such biases did not differ between any tested fiber types. Our findings provide key insights into fiber/bed interactions and transport pathways and imply that current monitoring methodologies significantly underestimate lotic (and potentially lentic) populations of fibers. We argue that it is crucial to sample for all fiber types, throughout the water column in all riverbed types, to understand fully the scale of riverine textile fiber pollution.

## Graphical abstract

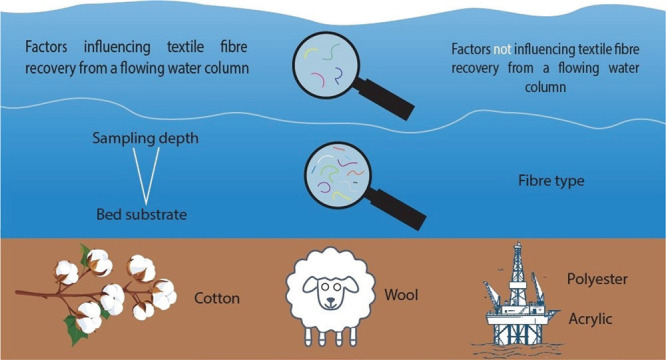

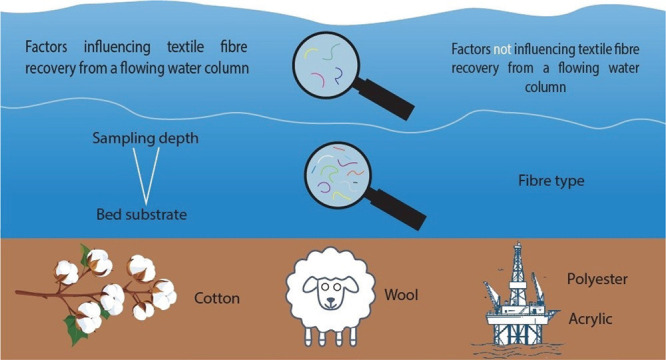

## 1 Introduction

Textile fibers, particularly those made from polymers such as polyester, polyamide (nylon), and acrylic, are frequently recorded as the most abundant particle morphology in microplastic surveys of aqueous environments.^1,2^ While plastic fibers have been well studied, nonplastic textile fibers (e.g., natural fibers such as cotton and wool and semisynthetic fibers such as viscose and lyocell) are highly abundant but have been largely neglected in anthropogenic microparticle research.^3,4^ Where entire environmental textile fiber populations (i.e., surveys that characterize all textile fiber types, not just plastic textile fibers) have been conducted, natural textile fibers, such as cotton and wool, have been found to dominate freshwater samples,^5^ as well as marine,^6^ atmospheric,^7^ and biotic^8^ samples. Collectively, environmental assessments of plastic and nonplastic textile fibers regularly refer to these pollutants as “microfibers”. Due to the technical definition of “microfibers” within the textile industry for synthetic yarns of specific diameter, not length,^9^ we refer only to textile fibers here.

Sources of textile fibers vary and include textile manufacture, laundering, and everyday wear.^10^ Depending on their source, the pathways of these fibers to aqueous environments also vary, with fibers derived from the laundering of garments and their subsequent movement to and through wastewater treatment plants being a particular focus of research for over a decade.^11^ With most riverine microplastic studies sampling only surface waters, the need for careful sample design throughout the depth profile of river systems has been identified as a key requirement for representative microplastic sampling.^12,13^ No similar work has incorporated natural textile fibers into assessments of sample design, with important implications for robust assessments of riverine textile fiber monitoring and the management, policy, and legislation this informs.

Plastic fibers can interact with biological and physical components of the environment. These fibers can act as vectors for organic and inorganic pollutants and be a source of chemical pollutants that leach from them.^14^ Plastic fibers can also be ingested by organisms across aquatic food chains.^15–20^ These fibers have been found to dominate wastewater,^21,22^ stormwater runoff,^23,24^ rivers,^25,26^ lakes,^22,27–29^ estuaries,^30–32^ and marine waters.^6,33–35^

While the assumed origin and biodegradability of natural fibers has often been taken to render their environmental impact limited, some studies have evaluated—and demonstrated—ecotoxicological impacts of natural textile fibers (e.g., Siddiqui et al.^36^). This work complements archeological evidence that natural textile fibers can persist over long periods of time in both aquatic (often associated with anoxia in peat, marine, and freshwater settings) and terrestrial (dry) contexts. For example, Chen and Jakes^37^ recovered a cotton waistcoat from a deep-sea shipwreck 133 years after it sank. Drivers of natural textile fiber persistence include the extensive processing that natural fibers undergo for textile applications. The mercerization of cotton (the most common natural textile fiber) changes the polymeric structure of cellulosic from cellulose I to cellulose II,^10,38,39^ a more thermodynamically stable compound.^40^ Moreover, the influence of dyes and finishing agents (softening agents, anti-wrinkle substances, and water repellents) can increase natural fiber durability.^41^ The addition of dyes and finishing agents, as well as the modification of natural fibers, means that they are “inherently unnatural” and present a potential direct source of chemical pollution, as well as sorbing and transporting other chemical pollutants.^42^ However, understanding transport pathways and mechanisms of all fiber types represents a key knowledge gap in this space.

The majority of plastic textile fiber research has focused on their presence in, pathways to and through, and impacts on aquatic environments. Within riverine systems, assessments of catchment-driven drivers of microplastic and textile fiber pollution are a common research focus (e.g., Xu et al.^43^ Tan et al.^44^). However, in addition to environmental factors, river sampling technique has the potential to introduce large amounts of uncertainty and error to environmental microplastic quantification (e.g., Cowger et al.^45^). It is not yet known if this applies to natural textile fibers as well.

Rivers are diverse in their morphologies, with features such as vegetation, woody material, riverbed grain size, structure and pool-riffle sequences, and anthropogenic modifications impacting river flow, river form (both plan and cross-section), and riverbed roughness.^46–48^ This, in turn, affects flow features such as turbulence,^49,50^ and subsequently the transport of suspended materials, including microplastics.^51–53^ Spherical microplastic particles have been observed to obey the same physical principles as natural particles, permitting numerical modeling of their transport depth profiles using particle Rouse numbers.^54–56^ However, differences in the morphologies of textile fibers, such as particle density (between-fiber type) and fiber length and curliness (within-fiber type), alter fiber settling velocity,^57^ meaning such variations in flow may differentially alter the transport of different fiber types.^52^ Investigating potential differences in textile fiber transport through the vertical profile of a flowing water column, accounting for different fiber types and riverbed morphologies, is therefore urgently needed to inform appropriate and reliable sampling and analysis strategies and to enable accurate inventories of fibers to be made.

Experimental assessments of the transport behaviors of microplastic particles have previously used flume facilities that mimic lotic environments, which can be manipulated to recreate specific flow conditions. The flow and settling velocities of various forms of microplastics have been investigated, identifying differences associated with particle morphology, density, weight, length, and surface-to-volume ratio, as well as polymer.^55,57–60^ Flume studies have also considered the influence of external factors, including vegetation,^61^ bed substrate,^62^ and large roughness elements^63^ on microplastic transport. Mechanisms of riverine microplastic transport, however, are largely understudied and where this has been assessed, findings are often contrasting.^64^ Flume experiments represent a key tool in advancing understanding of the behaviors of particulate pollutants in the environment, informing experimental design for field-based research and the appropriate interpretation of data thus generated. However, comparative assessments of natural and plastic textile fiber distributions are absent from current assessments of anthropogenic particle transport in lotic systems.

Here, we present a novel assessment of textile fiber transport behavior in a unidirectional flow experimental flume under typical turbulent flow conditions. We investigate the influence of bed surface morphology (substrate grain size) and textile fiber type on the distribution of textile fibers through a flowing water column, and we evaluate the suitability of surface-only sampling in riverine surveys of textile fiber pollution. We analyze differences across substrate grain size (flat, fine, and coarse) because of its key role in influencing flow hydraulics,^65,66^ and it is a variable routinely collected by environmental monitoring bodies (e.g., the Environment Agency^67^) and modeled at the landscape scale (e.g., Naura et al.^68^). In response to growing evidence that natural textile fibers dominate environmental samples, we compare this for four fiber types, two natural fibers (cotton and wool) and two petrochemical-based synthetic fibers (polyester and acrylic).

## 2 Methods

A series of laboratory flume experiments were undertaken, where the vertical distribution of fibers of four fiber types (cotton, wool, polyester, and acrylic) was recorded in flow over three bed roughness types (flatbed (glass flume channel base), fine gravels, and coarse gravels). Natural fiber types (cotton and wool) were confirmed to match fiber types stated on garment labels by optical microscopy, as per Stanton et al.^5^ Synthetic fiber types (polyester and acrylic) were confirmed to match the polymers stated on garment labels using FTIR spectroscopy.

### 2.1 Fiber Generation

Prior to experimental setup and garment selection, a blank (water only) run of the flume was used to collect samples of water and establish background textile fiber populations within the experimental setup. Textile fibers present in these blanks were predominantly gray-blue cotton fibers. This informed test garment selection, such that the color selection of the test fabrics did not reflect any of the fibers that had been identified in these blank runs.

Garments of 100% wool, cotton, polyester, and acrylic (determined by their labels and morphology (for cotton and wool as per Stanton et al.)^5^ and FTIR spectra (for polyester and acrylic)) were purchased from charity shops on the UK high street in 2023. Garments were chosen based on their distinctive color (informed by QA/QC procedures outlined below) to aid later identification. The chosen garments were a green cotton long-sleeve t-shirt, a brown wool jumper, a black polyester pair of sports shorts, and a red acrylic jumper Figure S1). The densities of these, as detailed in Morton and Hearle,^69^ increase in the order acrylic (1.19 g cm^–3^) < wool (1.30 g cm^–3^), polyester g cm^–3^) < cotton (1.55 g cm^–3^). A white laboratory coat was worn throughout the experimental procedure, and clothing matching the test fabrics was not worn.

Swatches of fabric were cut from each garment, with each swatch then cut at the edges with scissors over 5 glass 500 mL beakers. Each beaker contained 200 mL of deionized water, to which a mixture of all 4 fiber types was added. A magnetic stirrer was placed in each beaker, and the beakers were placed on a stirring plate for 30 s at 100 rpm to ensure all fiber types were suspended. For each experimental run (*n* = 15), a 20 mL subsample of water containing this fiber mixture was collected using a plastic syringe. A total of five 200 mL beakers were made, and so each beaker was used for three separate experimental runs.

The volume of water containing fibers that was introduced to the flume, and the size of the swatches used, were informed by extensive testing of these parameters to ensure sufficient fibers were released from the test garments, but not so many fibers that quantification of fibers captured became impractical. As the analysis conducted was determined by relative proportions of fibers collected across depths, the number of fibers introduced to the sample system did not need to be determined, and variability in the absolute number of fibers introduced to each run did not have an impact on the outcome of the experiments.

### 2.2 Determining Flume Conditions

Experiments were conducted in an Armfield S6 flow-recirculating flume that was 10 m long and 0.3 m wide. Bed substrate was installed into the flume using removable sediment boards, where either no sediment (hereafter “flat”), fine gravels (*D*_50_ = 9 mm), or coarse gravels (*D*_50_ = 30 mm) was fixed in place (following Mason et al.,^70^ Figure S2). Water depth (*h*, 0.33 m), flow velocity, and flume slope (0°) were kept constant, with flow velocity measured using a Valeport M002 electromagnetic flow meter. A coarse gravel board was positioned at the upstream end of the flume to ensure a uniform flow depth throughout the test section. Experiments were not hydraulically scaled (i.e., a 1:1 scale was employed), and flow was subcritical (Froude number = 0.084) and fully turbulent (Reynold’s = 16,109), with a shear velocity of 0.106 m s^–1^.

For each bed type, the experimental reach was determined by measuring the velocity of the water (at 0.4 depth from the bottom of the flume) at 0.5 m intervals along the entire length of the flume using two 30 s average flow readings at each 0.5 m interval. The experimental reach was determined as the length of the flume where uniform flow was observed (Table 1). Flow velocity between all runs, irrespective of bed type, varied by less than 0.025 m s^–1^ and a standard deviation of less than 0.02 m s^–1^.

**Table 1:** Flume Characteristics for Each Experimental Treatment

bed sediment type	median bed grain size (*D*_50_)	experimental reach length (m)	experimental reach location (m)	flow velocity (m s^–1^ ± 1 SD)
none (flat)	N/A	4.0	4.5 to 8.5	0.161 (±0.010)
fine gravel	9 mm	2.7	3.0 to 5.7	0.148 (±0.010)
coarse gravel	30 mm	3.0	3.5 to 6.5	0.145 (±0.011)

The experimental runs were devised to ensure the textile fibers had enough time to reach the nets (details below) before the nets were removed from the flume. With a run time of 25 s and an experimental reach of 4.0 m for the flatbed runs, an average flow rate of 0.161 m s^–1^ (the maximum used in this study) would have been sufficient for the fibers to travel 4.0 m. This experimental setup was evaluated in preliminary runs, where fibers were visually observed to populate the full vertical profile of the flume, indicating fibers could fully disperse in the experimental distance. Fibers captured in the bottom net during the experiments also demonstrate that there was sufficient time and distance for fibers to be distributed vertically throughout the water column. However, given the shorter experimental length, it is likely that fibers were not fully in equilibrium, with fibers likely overrepresented in the surface and middle nets. Thus, our results present a conservative estimate of the under-representation of fibers that surface-only sampling likely yields.

To limit the challenges presented by the accumulation of background debris (including fibers) within the recirculating flume, which could have hindered fiber quantification, a 10 μm zooplankton net (diameter 26 cm) was positioned upstream of the experimental reach. The purpose of this was to reduce the number of fibers collected in the nets during consecutive runs and, therefore, aid quantification of recovered fibers.

### 2.3 Flume Experiments

Five experimental runs were conducted for each bed type (*n* = 3), resulting in a total of 15 experimental runs. For each run, the mixture of textile fibers was released from a 20 mL syringe at a constant rate at the upstream end of the flume at the start of the experimental reach (Table 1 and Figure S3). The textile fibers were released at a syringe angle of ∼45° at a consistent rate just below the surface of the water to ensure fibers broke through the surface tension of the water. Textile fibers introduced to the flume were captured at the downstream end of the experimental length using circular nets positioned at three different sample depths: 0–11 cm (bottom of the water column), 12–23 cm (middle of the water column), and 24 cm surface (top of the water column; Figures 1 and S3). The nets were constructed for the purpose of the study, with an internal diameter of 10 cm and a net mesh aperture of 125 μm, and were held in place in the flume using a clamp stand. Preliminary investigations found finer mesh apertures introduced backflow within the net, inhibiting fiber collection. The nets were collected 25 s after the fibers were released from the syringe. During their removal from the flume, the clamp stand was lifted out of the water vertically and once out of the water tilted slightly backward, run times ranged from 10 to 15 min.

**Figure 1:**
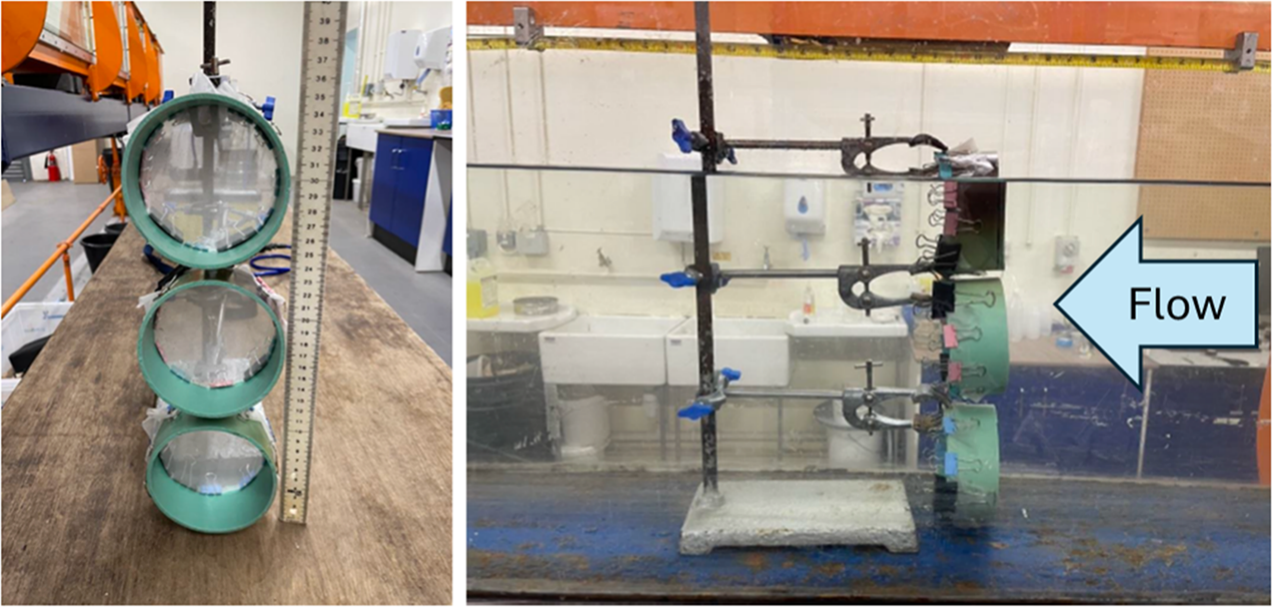
Positioning of the three nets used to capture fibers in the experimental set-up. The top net captured fibers from the water surface to a depth of 9 cm from the surface, the middle net 10 to 21 cm from the surface, and the bottom net 22 to 33 cm from the surface (the bed).

Each net was washed over a vacuum filtration apparatus using deionized water. Before filtering samples, the surfaces used were cleaned using paper towels to remove surface dust. All glassware, the vacuum filtration apparatus, rings, and nets were thoroughly rinsed with deionized water before each experimental run. Samples were vacuum filtered onto Whatman grade 1 qualitative circle 47 mm filter paper for analysis. The sides of the filter housing were washed thoroughly, and once filtered, the filter paper was transferred into a Petri dish, and the lid was sealed with electrical tape.

### 2.4 Microscope Analysis of Samples

Prior to the analysis of samples from experimental runs, familiarity with fiber morphology was established through observation of fibers taken from the garments used. Fibers from each garment were observed under a Zeiss Stemi 305 dissecting microscope (magnification range 8–40×), noting key features of cotton (flat, twisted ribbon) and wool (cuticles) fibers, as well as the shades of the chosen colors. As extruded fibers, color was the key distinguishing feature between polyester and acrylic fibers. Once confident in the appearance of each fiber type (Figure 2), experimental samples were visually analyzed using the same dissecting microscope. To reduce the likelihood of repeat-counting of fibers, each sample paper was analyzed in four quarters. Only fibers that matched the color and morphologies of the test fibers were quantified.

**Figure 2:**
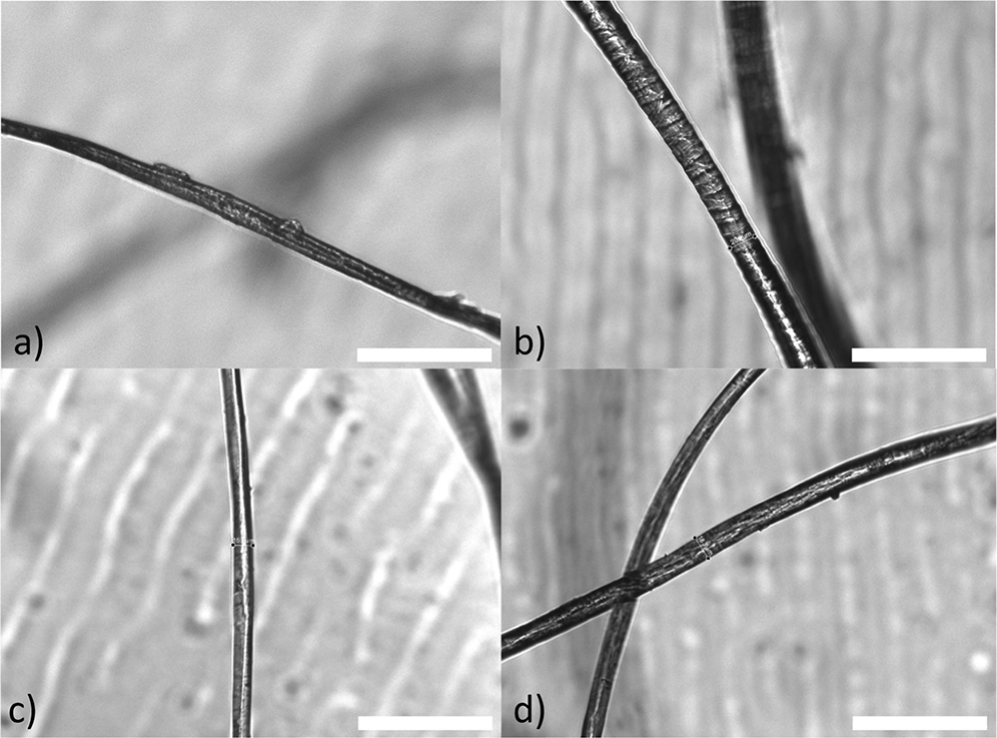
Micrographs of the four textile fiber types used in this study. Natural textile fibers have distinct morphologies, with cotton (a) resembling a flat twisted ribbon and wool (b) showing clear cuticles. Plastic fibers acrylic (c) and polyester (d) cannot be differentiated by morphology under brightfield microscopy and were differentiated by color (red and black, respectively) in this study (Figure S1). All scale bars represent 100 μm. Micrographs acquired using Agilent Biotek Cytation 1.

### 2.5 Statistical Analysis

All statistical analyses were undertaken in the R environment (version 4.4.2; R Core Team 2024). Differences in the distribution of textile fibers in the water column were tested using generalized linear models (GLM). The relative distributions of the textile fibers throughout the water column are reported and analyzed as proportions, rather than absolute values because (i) it was not possible to capture all fibers; (ii) the number of fibers introduced to the system was not quantified; (iii) the use of a recirculating flume means that some fibers introduced from previous runs could also be captured; and (iv) this provides a standardized scale for examining differences between fiber types.

Because most microplastic and textile fiber surveys of freshwater environments sample only from the surface of waterbodies, we first determined the proportion of fiber materials that were recovered from the surface water to evaluate the possible underestimation of textile fiber reporting using surface-only sampling strategies. We then analyzed whether the proportion of fibers recovered from the surface water (top net) compared to fibers recovered below the surface water (middle and bottom nets combined) was dependent on the fiber type, bed substrate, or an interaction of the two. A GLM fitted with a binomial error distribution and logit link structure using the “glm” function in the “stats” package was employed. The response variable was inputted as a matrix of the proportion of fibers captured in the top net and the proportion not in the top net. Posthoc pairwise comparisons were undertaken using estimated marginal means with *p*-values adjusted for multiple comparisons via Tukey tests within the “emmeans” package.^71^ Estimated marginal means were back transformed from the logit scale for presentation in the text.

Then, a second model examined the statistical difference between the distribution of textile fibers captured below the surface in the middle and bottom nets and whether this was dependent on the fiber type, bed substrate, or an interaction of the two. The response variable was inputted as a matrix of the proportion of fibers captured in the middle net and the proportion in the bottom net. A value of α = 0.05 was considered significant for all analyses.

## 3 Results and Discussion

### 3.1 Number of Textile Fibers Captured and Analyzed from the Flume Experiments

Across the 15 runs undertaken, a total of 18,793 fibers matching the test garments were counted. Most (8076; 42.9%) of these were acrylic, 4542 (24.2%) were wool, 4485 (23.9%) were cotton, and 1690 (9.0%) were polyester, with fibers of all types captured in all nets for all bed substrate types (Table 2). As data were analyzed using proportional distributions, disparities in the total number of fibers collected for each material do not impact the analysis and results, but these large numbers of fibers collected in all cases increase the precision and robustness of the results.

**Table 2:** Number of Fibers Collected in Each Experimental Run (Mean ± Standard Deviation), for Each Fiber Type under Each Substrate Condition

	flatbed	fine gravel	coarse gravel	sum
acrylic	416 ± 125	587 ± 149	612 ± 214	538 ± 179
polyester	73 ± 21	109 ± 31	155 ± 67	123 ± 54
wool	251 ± 57	303 ± 74	355 ± 137	303 ± 99
cotton	285 ± 84	262 ± 56	350 ± 123	299 ± 94
sum	256 ± 146	315 ± 195	368 ± 213	313 ± 189

### 3.2 Vertical Distribution of Textile Fibers within the Flowing Water Channel

#### 3.2.1 Surface versus Subsurface Fiber Transport

Across all runs, 26.5% (SE = 0.8%, min = 14.3%, max = 40.4%) of all fibers were recovered from the surface water net, indicating that in the described set-up, surface water only sampling would miss 73.5% of all fibers transported in the flow. The proportion of fibers captured in the surface water net did not differ between fiber type (Χ^2^ = 1.43, *p* = 0.70; acrylic = 27.3 ± 1%; polyester = 26.7 ± 1%; wool = 25.7 ± 1%; cotton = 25.7 ± 1%), indicating that surface water sampling underrepresents fibers equally.

The proportion of fibers in the surface water compared to below the surface varied significantly between bed types (Χ^2^ = 25.49, *p* < 0.001). Post hoc pairwise comparisons indicated that the proportion of fibers captured in the surface net with the coarse bed (30.5 ± 1%) was significantly greater than in the presence of the fine gravel (25.2 ± 1%) or flatbed (23.8 ± 1%), and there was no difference between the proportion of fibers captured in the surface water net between the fine gravel and flatbed (Figure 3a).

**Figure 3:**
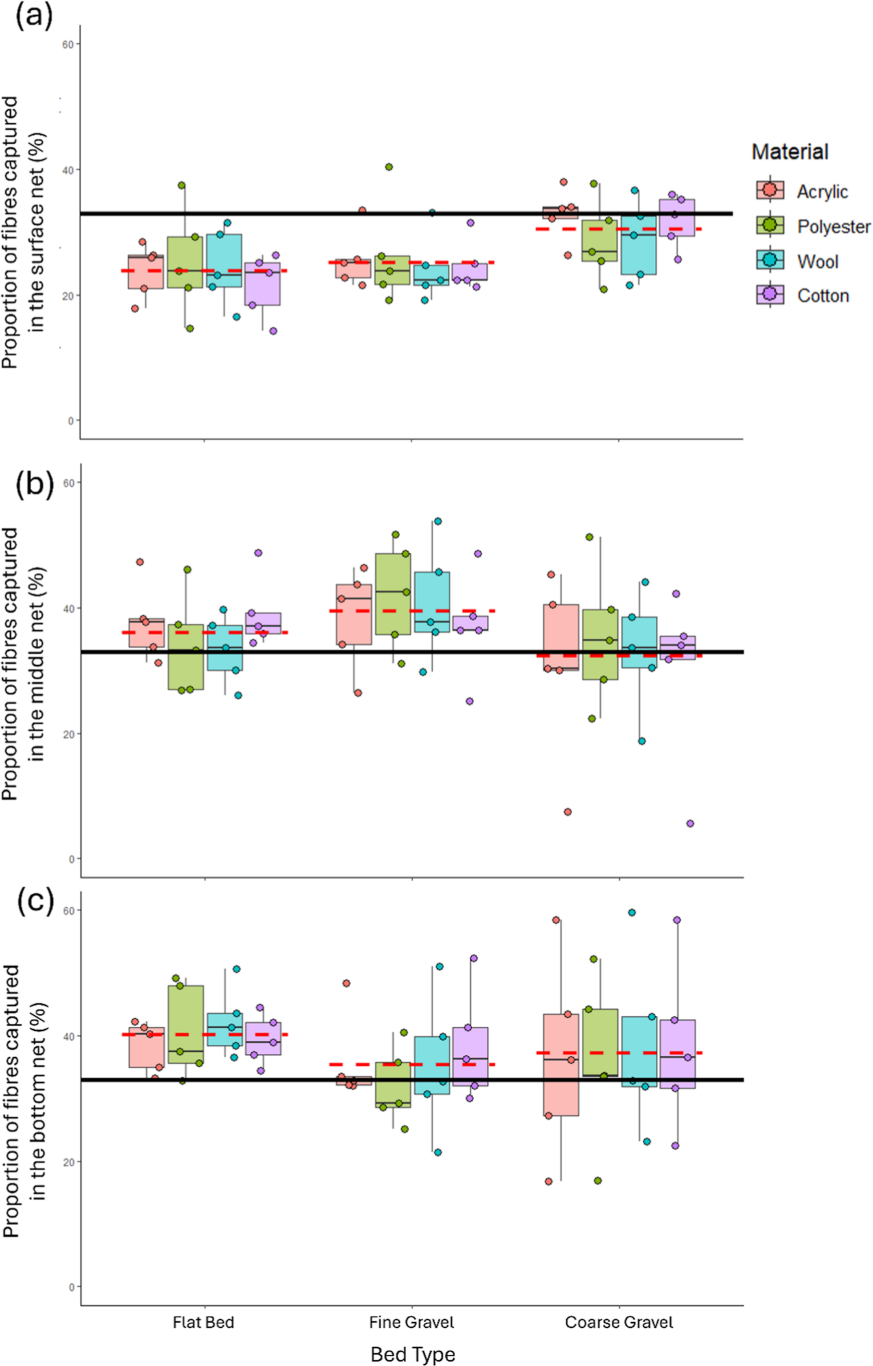
Distribution of fibers of each type (colors) across each bed type (clusters) in the (a) top, (b) middle, and (c) bottom nets. Black horizontal lines at 33% represent the proportion of fibers recovered if fiber distribution was equal throughout the water column. Red dotted line shows mean across fiber types for each substrate type.

There was no interaction between bed type and fiber type (Χ^2^ = 4.84, *p* = 0.57), indicating that while the proportion of all fibers trapped in the surface net varied with bed type, this variation was consistent across fiber type.

#### 3.2.2 Subsurface Fiber Transport

Across all runs, for fibers transported below the water surface, 48.9% (SE = 1.6%, min = 8.7%, max = 75.2%) were recovered from the middle net, and 51.1% (SE = 1.6%, min = 24.8%, max = 91.3%) were recovered from the bottom net.

The proportion of subsurface fibers recovered from the middle net compared to the bottom net again varied significantly between bed types (Χ^2^ = 18.57, *p* < 0.001). Post hoc pairwise comparisons indicated that the proportion of fibers captured in the middle net with the fine gravel bed (52.8 ± 1%) was significantly greater than in the presence of the coarse gravel bed (46.7 ± 1%) or flatbed (47.2 ± 1%), and there was no difference between the proportion of fibers captured in the surface water net between the coarse gravel and flatbed (Figure 3b,c).

The proportion of subsurface fibers captured in the middle net did not differ between fiber type (Χ^2^ = 3.23, *p* = 0.36; acrylic = 49.1 ± 1%; polyester = 50.7 ± 1%; wool = 48.4 ± 1%; cotton = 47.5 ± 1%). Again, there was no significant interaction between bed type and fiber type (Χ^2^ = 11.48, *p* = 0.07), indicating that while the proportion of subsurface fibers trapped in the middle and bottom nets varied with bed type, this variation was consistent across fiber type.

### 3.3 Surface-Only Sampling of Fibers as a Limited Approach

Our experiments show that the transport of two natural textile fiber types (cotton and wool) matches patterns from previous research showing that microplastic fibers are transported throughout the full water column.^72–74^ Surface-only sampling introduces considerable uncertainty and systematic bias in extrapolations of textile fiber abundance and flux. For plastic budgets, previous work has concluded that surface sampling protocols have the potential to underestimate plastic budgets by up to 90%.^55^ Here we find that, for our tested textile fiber types, 73.5% of the fibers recovered from our experimental setup were collected below the water’s surface. This pattern holds true independent of fiber type and bed substrate.

This work adds significantly to a body of research that has concluded depth-limited sampling protocols cannot be relied upon to generate microplastic data that are representative of sampled environments.^75^ Understanding the vertical distribution of textile fibers in rivers, as well as how fiber types are transported, deposited, and resuspended, is crucial to inform appropriate sampling strategies that can more accurately represent absolute and relative fiber concentrations and so total fiber inventories in aquatic environments.^2,76^

### 3.4 Surface-Only Sampling Biases Vary across Fiber Types, but Not Bed Morphologies

In this study, the under-representation of textile fibers by surface-only sampling and the influence of bed substrate on their vertical distribution through a unidirectional flowing water column were consistent across all tested fiber polymers. This is despite differing fiber morphologies (e.g., flat twisted ribbons of cotton, cuticle surfaces of wool, and smooth uniform profiles of extruded fiber; Figure 2), and differing polymer densities (acrylic 1.19 g cm^–3^; polyester 1.39 g cm^–3^; wool 1.30 g cm^–3^; and cotton 1.55 g cm^–3^ ^69^), with fibers of even the lowest density equally recovered from the lowest net. We therefore show for the first time that the same river sampling methods can be applied to recover natural and plastic textile fibers, with biases as a function of riverbed roughness being consistent across fiber types, meaning that natural fibers can, and should, easily be incorporated into future plastic fiber assessments. Further, the lack of differences in the mean proportional distributions of fibers suggests a general, first order correction factor for estimating total fiber loads from surface sampling could be developed for field application in the future.

However, our results also show that the total proportion of fibers captured using predominantly surface-only sampling methods shows bias depending on the bed substrate. We find that riverbed substrates with a low roughness show the greatest underestimation of fiber proportions and quantities. This pattern was likely driven by changes in flow turbulence as a result of the bed morphology. Concentration depth profiles of suspended particles in rivers are commonly predicted by the opposition of gravitational and turbulent forces, where particles with higher settling velocities relative to the water’s turbulent mixing are expected to display higher concentrations with depth.^54^ Given that the gravitational forces acting on the fibers were consistent between experimental runs, variation in the concentration depth profiles observed can be attributed to differences in turbulent forces. Turbulent mixing occurs due to hydraulic resistance in the channel, to which riverbed roughness, as a result of particle size, is a key determinant.^77,78^ The higher bed roughness associated with the gravel (over the sand and smooth bed) thus results in higher turbulence, increasing particle buoyancy and pushing a greater proportion of particles closer to the surface.

Such mechanisms have been modeled by previous researchers by applying the Rouse profile model to predict the vertical density distribution of microplastic particles in turbulent flows.^55,56^ However, while the application of the Rouse number has been well established for approximately spherical particles, assessments of fiber particles have found the length, curliness, orientation, and density of particles to change the settling velocity, by up to a 5.5-fold difference.^57,79–81^ Our fibers were generated in a way that yielded a variable range of length and curliness values, as was desired to reflect those found in the environment. At present, the typical distributions of length and curliness values for fibers are not known, and so at present, Rouse numbers cannot be robustly used for numerically modeling fiber distributions as they have been for spherical microplastic particles. However, studies have typically found microfibers to have settling velocities from 0.5 to 55 mm s^–1^,^57,80–82^ which can be used to calculate Rouse number *P* (eq 1). Tentative application of these settling velocities to the Rouse equation for our flume set up returned values of *P* = 0.0001 (0.5 mm s^–1^) to 0.013 (55 mm s^–1^), both centrally within the wash load parameters proposed by Rouse^54^ (0 < *P* < 0.8) and Cowger et al.^12^ (−0.8 < *P* < 0.8). Our experiments support these by demonstrating the majority of fibers were captured below the water surface as wash load but identify variation in the vertical distribution of fibers within the wash load. We therefore call for studies to characterize the morphological distributions and thus settling velocities of fiber populations in rivers to permit numerical modeling and computation fluid dynamic modeling of fiber transport depth profiles beyond those conditions examined in these experiments, to constrain specific correction factors for surface-only sampling in rivers of varying hydrology and geomorphology.

1 $$P=\frac{w_{s}}{\beta \kappa u^{*}}$$

where *P* is the Rouse number, *w*_s_ is the settling velocity, β is a factor correcting for eddy diffusion, usually set to 1, κ is the con Karman constant (0.41), and u* is the shear velocity.

Crucially, this under-representation of surface-only sampling of fiber populations identified in these experiments, and the underestimations associated with them, imply that without accounting for fluvial morphology and geomorphology, meaningful comparisons cannot be made within and between river catchments to understand the representative spatial distribution of textile fiber concentrations and transport pathways. Such bed grain size data can be rapidly visually estimated (e.g., River Habitat Survey), identified from national data sets, or can be estimated using spatial interpolation from known locations (e.g., Naura et al.;^68^ Sanders et al.^83^). As this study was undertaken at a consistent depth and flow velocity, variations of which will lead to different river hydraulics and thus alterations in vertical distribution, we do not present a quantified correction factor to apply for surface microfiber sampling (beyond first evidence that it may be an underestimate of the order of 4-fold), but highlight the importance of acknowledging bed substrate variability in understanding and upscaling surface fiber sampling. Now that biases in estimates using surface-only sampling have been identified to be a function of an easily quantified variable, further research quantifying interactive relationships between bed substrate, flow velocity, and flow depth should be pursued to create a quantified morphological and hydrological correction factor to permit accurate quantitative comparisons of transported textile fibers between locations and throughout time.

### 3.5 Application of Laboratory Results to Sampling in Rivers

While these experiments were undertaken in controlled laboratory flume conditions, the 1:1 scale employed and the flume dimensions make the results directly applicable to a wide array of real-world locations. First- and second-order streams comprise 77% of total global river length,^84^ and the depth used in the experiments (0.33 m) matches the mean depth (33.0 cm) of 9176 sampling locations across 4109 rivers routinely monitored by the Environment Agency of England and Wales—79% of which are dominated by fine sediment and gravel materials equivalent to those used in this study Environment Agency.^67^ Our results support in situ findings from three shallow (<0.5 m depth) urban gravel bed streams in Chicago, USA, where Vincent and Hollein^85^ found microplastic concentrations >100 times greater in the water column than in the stream surface. Further, our results are consistent with those from globally large rivers; Mendrik et al.^13^ reported that 86% of microplastics were transported below the water surface in the Mekong River (maximum >15 m depth), indicating that such subsurface transport pathways—and underestimation of total fiber flux from surface-sampling methodologies—are consistently observed across physical scales.

This work is of importance in a growing international body of research that is characterizing entire textile fiber populations (e.g., Stanton et al.;^5^ Suaria et al.;^6^ KeChi-Okafor et al.^86^). We support calls for monitoring efforts to sample throughout the entire depth profile of a river to gain a representative understanding of the relative concentrations of all textile fiber types^12^ and to support, challenge, and extend experimentally derived correction factors for surface-only sampling. We recognize that resource and logistical limitations mean that sampling throughout the depth profile of a river is not possible for all studies. However, riverine microplastic concentrations are highly variable—environmental assessments of microplastic variability have shown that flux calculations from surface water samples can vary by up to 8 orders of magnitude.^87^ Incorporation of such survey efforts will better constrain the errors of any associated extrapolation from riverine textile fiber quantifications or may indicate whether such extrapolations are appropriate. We therefore stress the need for riverine textile fiber surveys to discuss their findings in the context of their limitations, key to which is not only the point within the depth profile of a river where the samples were collected but also the fluvial morphology and geomorphology.

## 4 Conclusion

Natural textile fibers are widely promoted as greener alternatives to plastic textile fibers. However, natural fibers, like synthetic fibers, are the product of multiple environmentally hazardous anthropogenic processes, making them inherently unnatural, and thus, there is a pressing need to understand the pathways and impacts of these particulate pollutants to confidently establish the extent of their dominance. Here, we show that the majority (∼75%) of textile fibers travel through flowing waterbodies below the water surface and that while all observed processes were consistent across tested fiber types, bed substrate significantly influences the distribution of natural and plastic fibers in flowing water. Therefore, sampling strategies must acknowledge the environmental conditions in which they were taken, guided by the findings presented here. Building on this, the consistency of these preliminary results across fiber types and bed substrates gives some support that predictive correction factors may be possible to estimate from experimental and field observations and applicable across a range of real-world rivers. This would potentially have major implications for quantifying microplastic and fiber loads for monitoring, understanding, and managing environmental pollution and its impacts in aquatic systems.

## Supplementary Material



## Notes

Additional experimental details, materials, and methods, including photographs of experimental setup (PDF)
