# Supplementary material for: Flowing Fibers: Subsurface Sampling Is Key to Understanding Natural and Plastic Textile Fiber Pollution in Rivers
Source: ACS ES T Water. 2025 Dec 29;6(1):263–72. doi: 10.1021/acsestwater.5c00998 (PMC12797243; doi:10.1021/acsestwater.5c00998)
Supplement: Supplementary file 1 [file ew5c00998_si_002.pdf]

Flowing Fibres: subsurface sampling is key to understanding natural *and* plastic textile fibre pollution in rivers

Harley Nicholls[1,2], Catherine Sanders[3], David B. Ryves[1], Edwin Baynes[1], Kelly J. Sheridan[4], and Thomas Stanton[1*]

1. Department of Geography and Environment, Loughborough University, Loughborough, LE11 3TU, United Kingdom.
2. Environment Agency (EA), Department for Environment, Food and Rural Affairs (Defra), Bristol BS1 5AH, United Kingdom
3. University of Lincoln, School of Natural Sciences, Brayford Way, Brayford Pool, Lincoln, LN6 7TS, United Kingdom.
4. Department of Applied Sciences, Faculty of Health and Life Sciences, Northumbria University, Newcastle Upon Tyne, NE1 8ST, United Kingdom.

Supplementary Information

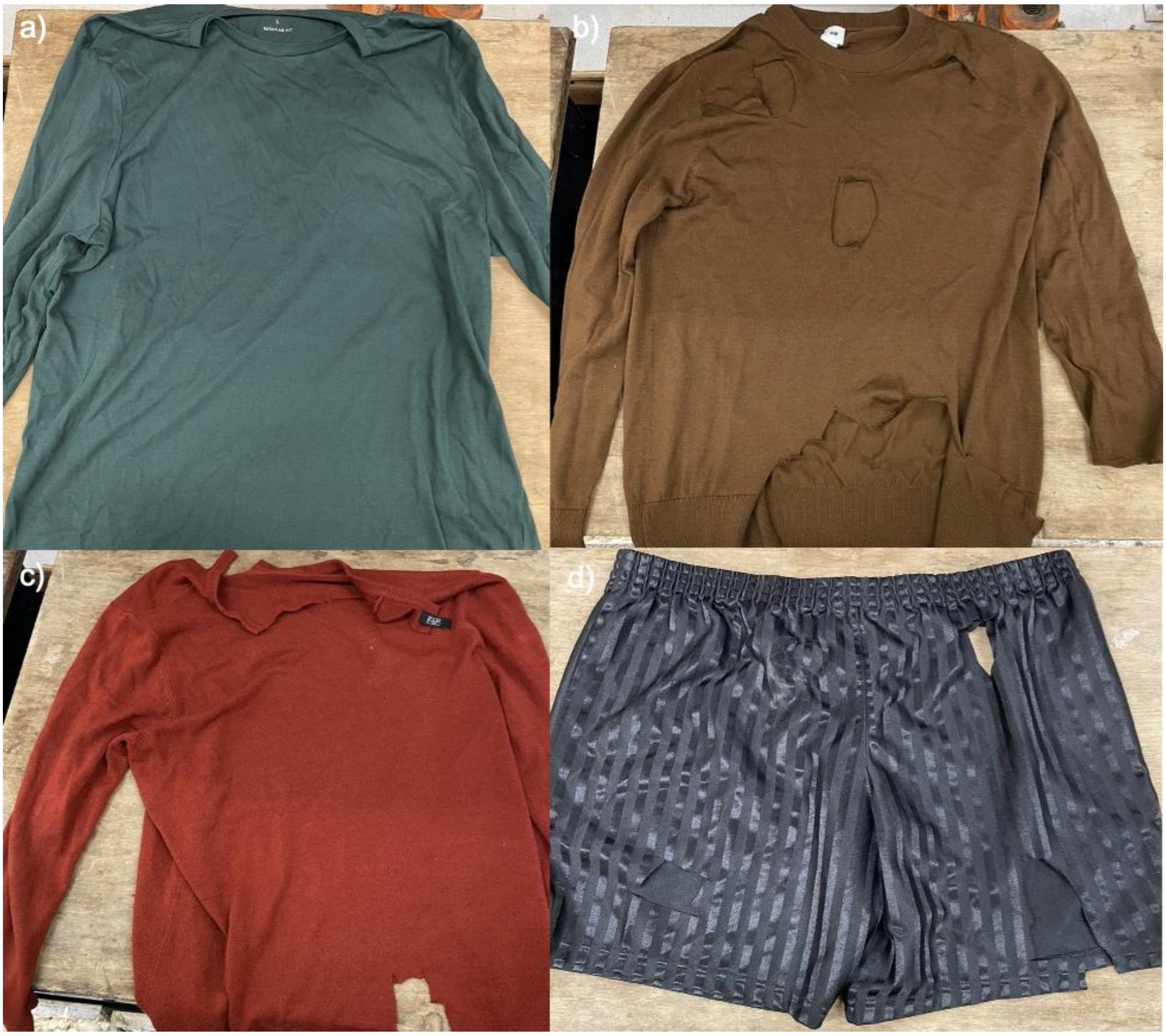

Figure S1. The clothing items used to generate the microfibres. (A) Cotton (B) Wool (C) Acrylic (D) Polyester.

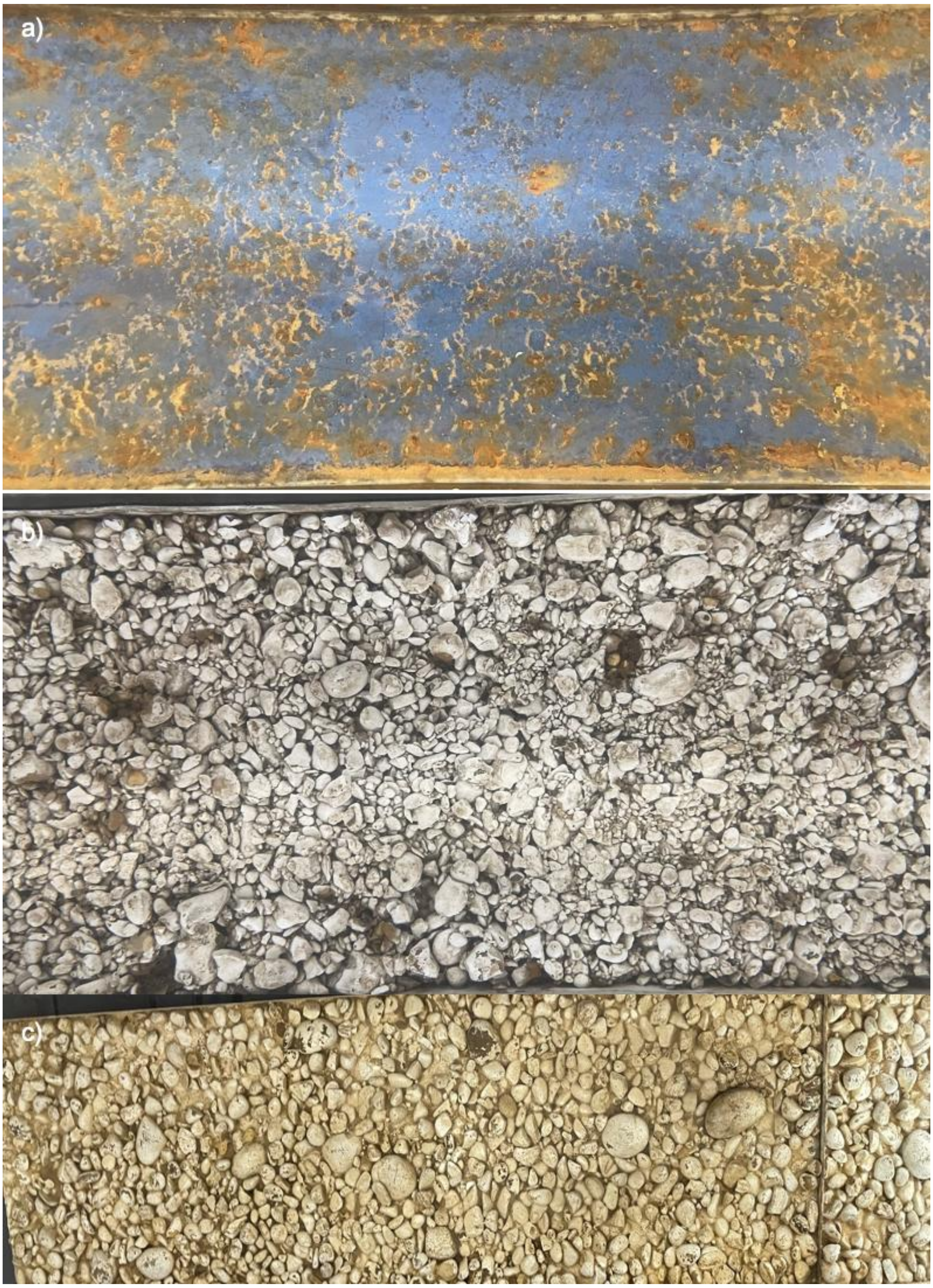

Figure S2. The three-bed types used (A) Flat bed (B) Fine gravel bed (C) Coarse gravel bed

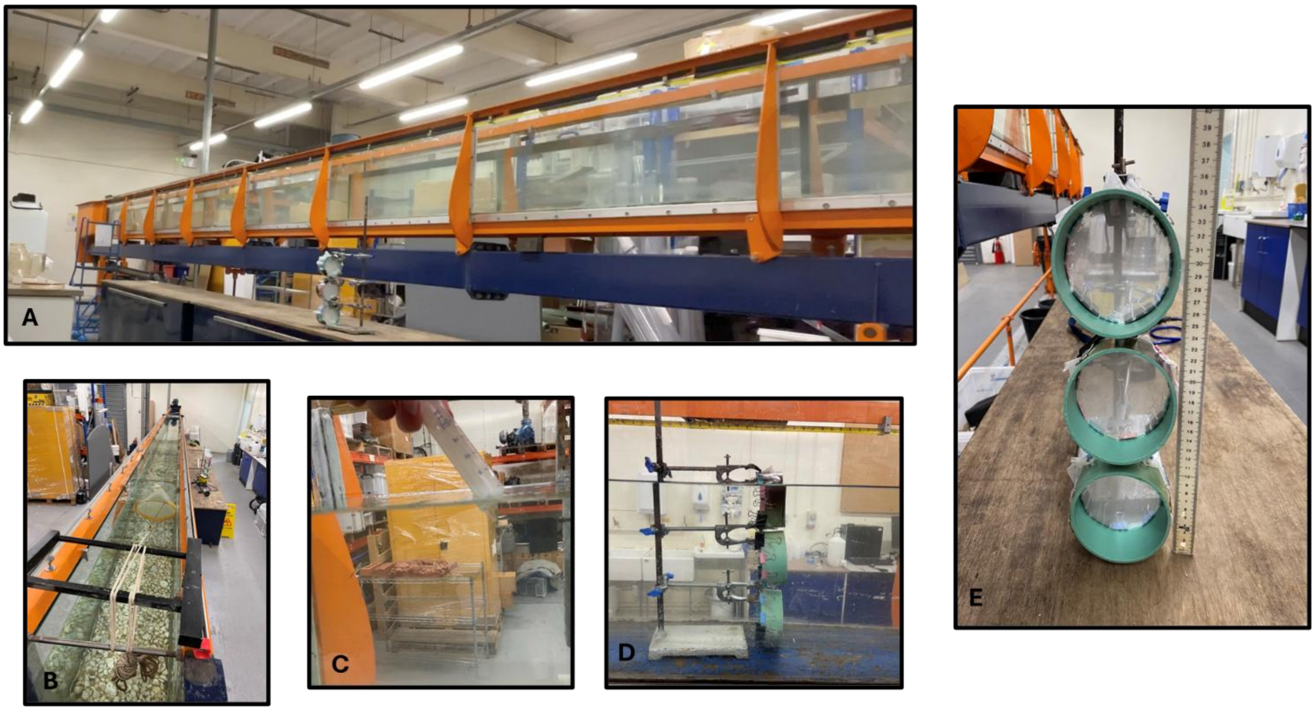

Figure S3. Photographs of experimental set up showing (A) the experimental flume used;' (B) the deployment of a net to reduce background debris in the water column; (C) the introduction of textile fibres to flume; (D) nets deployed in the flume; and (E) nets arranged vertically (outside of the flume).
